# Characterization of Equine Parvovirus in Thoroughbred Breeding Horses from Germany

**DOI:** 10.3390/v11100965

**Published:** 2019-10-18

**Authors:** Toni Luise Meister, Birthe Tegtmeyer, Yannick Brüggemann, Harald Sieme, Karsten Feige, Daniel Todt, Alexander Stang, Jessika-M.V. Cavalleri, Eike Steinmann

**Affiliations:** 1 Department of Molecular and Medical Virology, Faculty of Medicine, Ruhr-University Bochum, 44801 Bochum, Germany; Toni.meister@rub.de (T.L.M.); Yannick.brueggemann@rub.de (Y.B.); Daniel.todt@rub.de (D.T.); Alexander.stang@rub.de (A.S.); 2 Institute for Experimental Virology, TWINCORE Centre for Experimental and Clinical Infection Research; a Joint Venture between the Medical School Hannover (MHH) and the Helmholtz Centre for Infection Research (HZI), 30625 Hannover, Germany; Birthe.tegtmeyer@twincore.de; 3 Institute of Virology; University of Veterinary Medicine Hannover, 30559 Hannover, Germany; Harald.sieme@tiho-hannover.de (H.S.); Karsten.feige@tiho-hannover.de (K.F.); 4 Department for Companion Animals and Horses, University of Veterinary Medicine, 1210 Vienna, Austria

**Keywords:** equine parvovirus-hepatitis, Germany, risk factors, transmission

## Abstract

An equine parvovirus-hepatitis (EqPV-H) has been recently identified in association with equine serum hepatitis, also known as Theiler’s disease. The disease was first described by Arnold Theiler in 1918 and is often observed with parenteral use of blood products in equines. However, natural ways of viral circulation and potential risk factors for transmission still remain unknown. In this study, we investigated the occurrence of EqPV-H infections in Thoroughbred horses in northern and western Germany and aimed to identify potential risk factors associated with viral infections. A total of 392 Thoroughbreds broodmares and stallions were evaluated cross-sectionally for the presence of anti-EqPV-H antibodies and EqPV-H DNA using a luciferase immunoprecipitation assay (LIPS) and a quantitative PCR, respectively. In addition, data regarding age, stud farm, breeding history, and international transportation history of each horse were collected and analysed. An occurrence of 7% EqPV-H DNA positive and 35% seropositive horses was observed in this study cohort. The systematic analysis of risk factors revealed that age, especially in the group of 11–15-year-old horses, and breeding history were potential risk factors that can influence the rate of EqPV-H infections. Subsequent phylogenetic analysis showed a high similarity on nucleotide level within the sequenced Thoroughbred samples. In conclusion, this study demonstrates circulating EqPV-H infections in Thoroughbred horses from central Europe and revealed age and breeding history as risk factors for EqPV-H infections.

## 1. Introduction

Equine serum hepatitis (i.e., Theiler’s disease (TD)) is a serious and potentially life-threatening disease and one of the most common causes of acute hepatitis and liver failure in horses [1]. Specific treatment options are still lacking. TD was first reported in 1918 by Sir Arnold Theiler, after he observed signs of liver disease in animals vaccinated against African horse sickness with a combination of live virus and equine antiserum [2]. Similar to historical outbreaks of human posttransfusion hepatitis, multiple outbreaks of Theiler´s disease have been observed following parenterally administered equine serum products [3,4,5,6]. An incidence between 1.4%–18% of fulminant hepatitis among horses receiving an equine biological product has been reported [2,7]. Given the association between prior treatment with equine serum/plasma and the appearance of Theiler’s disease an etiologic role for a contaminating toxin or infectious agent has been suggested [8]. However, the exact pathogenic agent remained unknown for nearly a century.

*Parvoviridae* comprise a large family of non-enveloped DNA-viruses, which is currently subdivided into eight genera collectively known as parvoviruses. Members of this family have been described to infect a wide array of hosts, including humans, domestic, and wild animals [9,10,11]. Parvovirus infections have been associated with various severe and fatal diseases affecting the respiratory, gastrointestinal, and haematological systems and further potentially causing abortions [6,12,13,14]. Most recently, a novel equine Parvovirus (equine parvovirus-hepatitis virus (EqPV-H)) was isolated from serum and liver tissue of a horse that died of TD following administration of tetanus antitoxin (TAT) [15]. Administration of TAT contaminated with EqPV-H further resulted in seroconversion and acute hepatitis in experimentally infected horses, indicating that EqPV-H might be the causative agent of TD [15,16]. A recent study further reported a high prevalence of EqPV-H among commercial equine serum pools, indicating the necessity of careful risk assessment for medical and research applications [17]. However, despite its association with equine diseases, EqPV-H has not gained much attention of equine veterinarians and its worldwide prevalence and epidemiology remain poorly investigated.

Here, we examined the prevalence of EqPV-H among Thoroughbred breeding horses in northern and western Germany to identify potential risk factors for EqPV-H infections. A total of 392 serum samples from Thoroughbred broodmares and stallions were analysed for the presence of anti-EqPV-H antibodies, DNA, and viral sequences, respectively. Furthermore, an analysis of risk factors potentially affecting the prevalence of EqPV-H infections was performed to investigate natural routes of virus transmission.

## 2. Materials and Methods

### 2.1. Serum Sample Collection

A total of 392 serum samples from Thoroughbreds stabled on stud farms in northern and western Germany (Lower Saxony, North Rhine-Westphalia, Hamburg, Schleswig-Holstein) representing more than 25% of all registered breeding horses at The German Thoroughbred Studbook Authority (Cologne) were collected and processed between 2012 and 2015 [18]. All samples were then stored at −80 °C until further analysis regarding the presence of EqPV-H. 

### 2.2. Detection of EqPV-H DNA

Viral DNA was extracted with a viral DNA Kit from Qiagen (Cat. No. 1048147, Hilden, Germany) according to the manufacturer’s recommendations. DNA samples were stored at −20 °C until further analysis. A probe-based quantitative polymerase chain reaction (qPCR) was used with primers and probe designed and provided by Dr. Amit Kapoor as described previously [15]. A serial dilution of a plasmid containing the EqPV-H VP1 sequence was generated as standard row for the quantification of EqPV-H within the samples tested. qPCR measurements were performed using the LightCycler 480 real-time PCR system (Roche, Mannheim, Germany).

### 2.3. Detection of Anti-EqPV-H Antibodies

Samples were analysed for the presence of anti-EqPV-H-VP1 antibodies using the previously described luciferase immunoprecipitation system (LIPS) [19,20,21]. The EqPV-H-LIPS antigen VP1 was produced as described by Divers et al. [15]. Following the LIPS assay, relative light units (RLU) were determined using a plate luminometer (LB 960 XS3; Berthold, Bad Wildbad, Germany). To calculate sensitivity the mean RLU plus three standard deviations (SD) of an EqPV-H negative horse serum was defined as a cut-off limit. A potential cross-reactivity between the LIPS and other related parvoviruses could not be excluded.

### 2.4. Data Collection and Study Design

Three different groups regarding the state of EqPV-H infection were distinguished: Seropositive and EqPV-H DNA positive (DNA^+^/AB^+^), seronegative and EqPV-H DNA negative (DNA^−^/AB^−^), and seropositive and EqPV-H DNA negative (DNA^−^/AB^+^). Information regarding gender, age, state of reproduction, breeding, and international transportation history of the study population were received earlier from the Association for breeding and racing of Thoroughbreds (Cologne, Germany). Furthermore, the established criteria were subdivided into different groups. Age groups of similar size were created: 3–6-year-old horses (*n* = 78), 7–10-year-old horses (*n* = 113), 11–15-year-old horses (*n* = 129), and 16–29-year-old horses (*n* = 72). Similarly, horses were assigned to groups regarding the breeding history (0–4 breeding years) and the stock size of the stud farms (1–9 horses, 10–39 horses and >40 horses). Additional information about the transportation history was sorted according to the target country (France, Great Britain, Ireland, Germany, etc.). The data were determined by the time of sample collection between 2012–2015.

### 2.5. Sequencing and Phylogeny

For sequence analysis, a PCR (I) was designed within the NS1 of EqPV-H [17]. PCR was performed using the expand high fidelity PCR system (Roche Diagnostics, Basel, Switzerland) as described before [17]. PCR products were visualised on a 2% agarose gel, excised, and purified using a Monarch^®^ DNA gel extraction kit (New England Biolabs, Ipswich, Massachusetts, United States). Purified products were then sent for Sanger sequencing using the applicable PCR primers. Highlighter plot analysis [22] displaying nucleotide exchanges in EqPV NS1 in the screened cohort compared to a previously published strain from Europe (MK792434). Labelling of nucleotides according to IUPAC code. Length of bar scale of neighbour-joining tree indicating number of nucleotide exchanges. The input multiple sequence alignment was created with Mega X.

## 3. Results

### 3.1. Frequent Occurrence of EqPV-H among German Thoroughbreds

**Figure 1:**
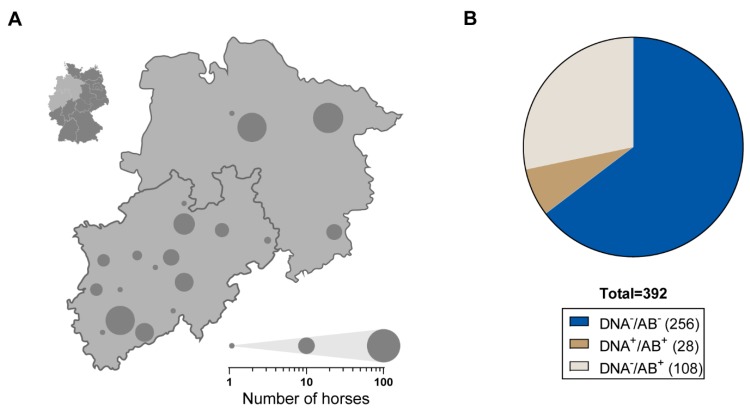
(**A**) Map of Germany highlighting the location of sampling (insert upper left). Serum samples were collected from 392 Thoroughbreds in North Rhine-Westphalia and Lower Saxony. Grey circles highlight the sample location and circle size is scaled to relative the number of examined horses (see legend lower right). (**B**) Serum samples were analysed for the presence of anti-equine parvovirus-hepatitis (anti-EqPV-H) VP1 AB and EqPV-H DNA performing luciferase immunoprecipitation system (LIPS) and qPCR, respectively. Individual horses were assigned to three different groups: Seronegative and EqPV-H DNA negative (DNA^−^/AB^−^; 65.3%), seropositive and EqPV-H DNA negative (DNA^−^/AB^+^; 27.55%), and seropositive and EqPV-H DNA positive (DNA^+^/AB^+^; 7.14%).

We first investigated the frequency of EqPV-H infections among Thoroughbreds from the north and west of Germany. A total of 392 serum samples from Thoroughbreds originating from Lower Saxony and North Rhine-Westphalia were collected during an annual fertility monitoring and tested for the presence of anti-EqPV-H-VP1 antibodies and EqPV-H DNA, respectively (Figure 1A). A total of 28 samples were tested positive for EqPV-H DNA (7.14%) via qPCR. We further evaluated the samples for the presence of anti-VP1-antibodies using a previously described LIPS [17]. An elevated serum titre of anti-EqPV-H-VP1 antibodies above the detection limit was present in 136 (34.69%) horses. Based on these findings the individual horses were assigned to the following groups: DNA^−^/AB^−^; DNA^−^/AB^+^ and DNA^+^/AB^+^ (Figure 1B). Interestingly, no sample was positive for EqPV-H DNA only, indicating no acute EqPV-H infections within the examined cohort.

### 3.2. Viral Characteristics of EqPV-H-Positive Horses

**Figure 2:**
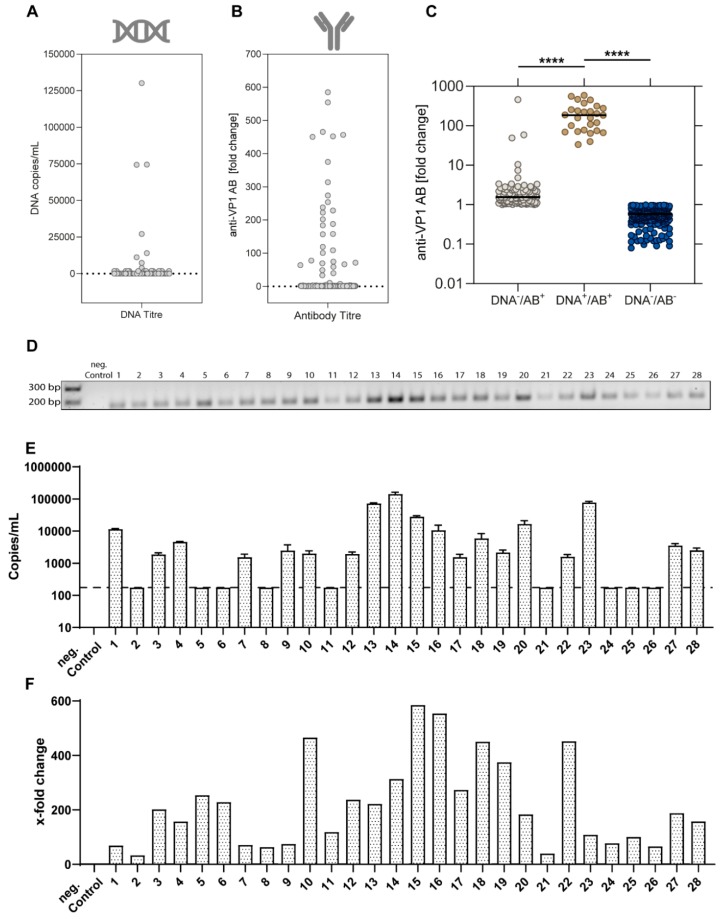
(**A**) Viral loads of EqPV-H (DNA copies/mL) were determined via qPCR (*n* = 392). Horses with viral load of >175 copies/mL were considered EqPV-H DNA positive. (**B**) EqPV-H VP1 antibodies were detected using a LIPS assay and the relative increase of RLU compared to an EqPV-H-negative control sample was calculated (*n* = 392). (**C**) Comparison of relative EqPV-H VP1 antibody levels between different groups: (Median values; DNA^−^/AB^−^, *n* = 256; DNA^−^/AB^+^, *n* = 108; DNA^+^/AB^+^, *n* = 28). Statistical significance was determined using a one-way ANOVA with Dunnett’s post-hoc test. (**** *p* < 0.0001). (**D**) Agarose gel electrophoresis of the qPCR product from DNA positive serum samples (*n* = 28) and an EqPV-H-negative control sample. (**E**) Viral loads of EqPV-H as determined by qPCR are displayed in DNA copies/mL. The dotted line indicates the limit of detection (mean +/− SD, *n* = 3). (**F**) Evaluation of serum titres for anti-EqPV-H antibodies in EqPV-H DNA positive serum samples determined by LIPS. RLU was normalised to the cut-off limit and is displayed as x-fold change.

Next, we characterized the distribution of viral loads in the study cohort showing three high-titre samples with copy numbers above 5 × 10^4^ DNA copies/mL (Figure 2A). For the anti-EqPV-H-VP1 antibodies, titres up to a 600-fold increase over the limit of detection were observed (Figure 2B). Interestingly, DNA^+^/AB^+^ samples showed significantly higher titres of anti-VP1-antibodies as compared to the DNA^−^/AB^+^ horses (Figure 2C). The 28 EqPV-H positive samples were further confirmed in a gel electrophoresis of the qPCR products (Figure 2D). The highest viral loads were detected for samples 13, 14, 15, 20, and 23, respectively (Figure 2E). The highest anti-VP1-antibody levels were observed for samples 10, 15, 16, 18, and 23 (Figure 2F).

### 3.3. Age and Breeding History are Potential Risk Factors of EqPV-H Infection in Thoroughbreds

**Figure 3:**
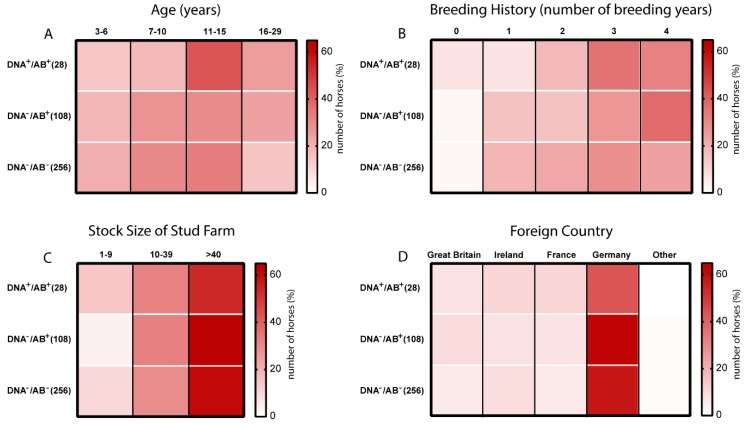
Heatmaps displaying the occurrence of anti-EqPV-H VP1 AB and EqPV-H DNA with regard to potential risk factors. The three groups based on the EqPV-H infection status were further classified based on (**A**) age, (**B**) breeding history, (**C**) stock size of the stud farm, and (**D**) foreign country. The colouring indicates the number of horses (in %) classified in the specific category. Each row adds up to 100%.

We next performed an analysis using data on age, breeding history, stock size of the stud farm, and transportation to a foreign country to determine factors, which might be involved in promoting EqPV-H and would identify natural routes of transmission. All results of the descriptive analysis are shown in Figure 3, with detailed information provided in Table 1. By analysing the impact of the age on EqPV-H infection, it became apparent that with increasing age, especially between 11–15 years, the proportion of EqPV-H DNA positive and seropositive horses [DNA^+^/AB^+^] was elevated (Figure 3A). In the group of 3–6-year-old horses approximately 14% were tested DNA positive and seropositive as compared to 43% for the group of 11–15 and 25% for the group of 16–29-year-old horses (Figure 3A). Likewise, a slight increase in the fraction of seropositive horses was observed upon increasing breeding history (Figure 3B). Regarding the classification of the stud farm based on their stock size, no difference between the different groups could be noted (Figure 3C). More horses were recruited from farms with more than 40 horses (Table 1). Most of the sampled and analysed horses stayed in Germany and other countries including Great Britain (*n* = 27), Ireland (*n* = 33), and France (*n* = 26). Importantly, travelling history to another country was not associated with an increased risk for EqPV-H infection (Figure 3D and Table 1). 

In a recent study, we observed a frequent occurrence of equine hepacivirus (EqHV) infections among the same cohort of Thoroughbreds with approximately 62% seropositive horses for EqHV antibodies [18]. We surveyed whether an EqPV-H co-infection potentially favours an EqHV infection. However, a similar prevalence of approximately 60% seropositivity for EqHV was observed among all groups of horses, indicating that an EqHV infection does not predispose individual horses for an EqPV-H infection (Table 1 and Figure S1). Overall, we observed an age dependent state of EqPV-H infection and our results further suggest a slightly higher prevalence among horses with an extended breeding history. In contrast, no correlation between the stock size of the stud farm and transportation history to a foreign country could be observed indicating that neither acts as a predisposing factor for an EqPV-H infection.

### 3.4. Sequence and Phylogenetic Analysis of EqPV-H Detected in Thoroughbreds

**Figure 4:**
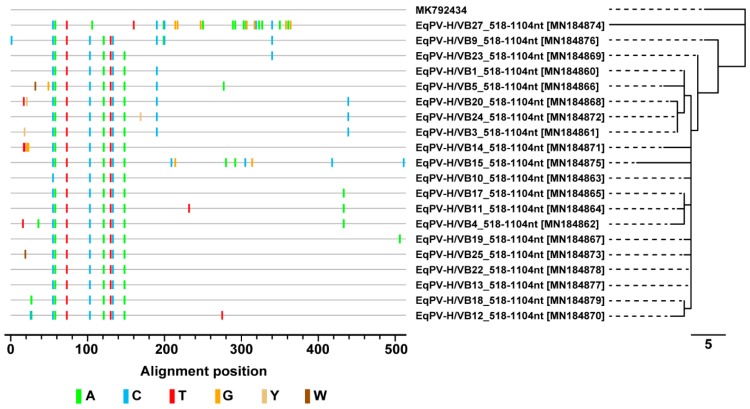
Highlighter plot displaying nucleotide exchanges in EqPV NS1 in the screened cohort compared to a previously published strain from Europe (MK792434). Labelling of nucleotides according to the International Union of Pure and Applied Chemistry (IUPAC) code. Length of bar scale of neighbour-joining tree indicating number of nucleotide exchanges. The input multiple sequence alignment was created with Mega X.

We next performed a molecular characterization of the EqPV-H DNA positive samples. A PCR (I) was designed to amplify a region within the NS1 gene of EqPV-H and the obtained amplicons were subjected to conventional Sanger sequencing. The obtained sequences were submitted to the GenBank database with the accession numbers indicated in Table 2. Due to very low viral loads sequencing reactions could not be successfully performed for all samples (20/28 sequences for PCRI). The obtained sequences were highly similar to a previously described EqPV-H sequence (Figure 4). As indicated by the highlighter plot and the length of the tree branches, a high grade of genomic conservation is found within the analysed cohort. Sequence identity with the previously described European strain is ranging from 95% to 98% (Figure 4). In accordance with previous findings these results indicate a high degree of conservation and genomic stability between the world-wide circulating strains and low genetic variability of the EqPV-H strain.

## 4. Discussion

In this cross-sectional study, we examined the prevalence of EqPV-H among almost 400 Thoroughbreds in northern and western Germany representing the first analysed European country. Based on the information from the German Association for breeding and racing of Thoroughbreds (Cologne), a total of 1450 brood mares and about 80 stallions were registered as breeding horses in 2014. Thus, we investigated about a quarter of the actively breeding Thoroughbreds in Germany and the results can be considered representative for the region. We observed a frequent occurrence of EqPV-H infections with 34.69% seropositive and 7.14% viraemic horses. The phylogenetic analysis demonstrated a high level of conservation in NS1 sequences in comparison with the world-wide circulating strains implying low levels of genetic variability. A detection of EqPV-H DNA with concurrent absence of anti-EqPV-H-VP1 antibodies was not observed for any sample, indicating no acute infection at the time of sampling. Of note, a cross-reaction in the LIPS assay with other potential parvoviruses in the horse samples cannot fully be excluded. Previously, in the first report describing the identification of EqPV-H a PCR prevalence in serum of 13% (13/100) was reported in the USA [15], and in China the average prevalence was 11.9% (17/143) in racehorses [11]. Further prevalence studies with larger cohorts from various continents are required for a more detailed epidemiology of EqPV-H.

We also performed a descriptive comparison between potential risk factors affecting EqPV-H prevalence. Iatrogenic transmission of EqPV-H has been demonstrated by Divers et al. through the administration of equine origin blood products [15], which should be confirmed ideally with a clonal viral EqPV-H inoculum in future studies. Of note, the virus can also be transmitted to horses that did not have such treatments [8]. The relative number of viraemic and seropositive horses in our analysis was increased with advanced age, especially between 11 and 15 years of age, but also in the group of 16–29-year-old horses compared to DNA^−^/AB^−^ and DNA^-^/AB^+^ horses. Possible reasons can be persistent humoral immunity or high risk of re-infection with longer living times. Due to the study design, horses younger than three years were not included. Therefore, it is impossible to draw a conclusion whether young foals in which the immune system is still maturing are at an increased risk of infection. Stage of reproduction was additionally accompanied by slightly higher fraction of EqPV-H infections. However, given that an extended breeding history mostly involves an advanced age of the examined horse this observation might be due to an indirect correlation. Neither stock size of the stud farm, transportation history to a foreign country, or an EqHV infection could be identified as a predisposing factor.

Of note, our previous investigation of the same study cohort revealed that younger Thoroughbreds transported abroad were at a higher risk to get infected with EqHV than horses at the same age staying in Germany. It is described that transportation of horses [23,24,25] and a changing environment as well as disruption of established social groups [26] can lead to a stress-induced immunosuppression, which could favour viral transmission. This risk factor seems therefore not to be highly involved in the susceptibility of horses to EqPV-H infections. Furthermore, equine hepacivirus co-infection was not a determinant for increased EqPV-H infections. Recently, a high rate of co-infections of these two equine viruses was also described in the prevalence report by Lu et al. from China [11]. As an additional way of viral spreading, we also investigated the potential of vertical transmission of EqPV-H in a single case. Postnatal serum samples were taken from a foal after delivery from an EqPV-H DNA positive and anti-VP1-positive mare (Figure S2). Transfer of EqPV-H-specific antibodies to the foal via colostrum could be observed, but no viremia was detected. The specific antibody transfer was expected, since the colostrum represents the important route of antibody transfer in neonatal foals, whereas the placental barrier hampers intrauterine transfer of macromolecules such as immunoglobulins from the mare to the foetus [27]. 

In conclusion, these data revealed that Thoroughbreds from central Europe have a frequent occurrence of anti-VP1 antibodies and EqPV-H DNA suggesting circulating EqPV-H infections in the horse population, endemic herds, or persistent shedding. In addition, age and multiple breeding were identified as potential risk factors for EqPV-H transmission.

## Figures and Tables

**Table 1:** General information regarding gender, age, breeding history, stock size, travel background, and EqHV-coinfection of the equine serum samples collected from Thoroughbreds in North Rhine-Westphalia and Lower Saxony.

Variables (*n*)	State of EqPV-H Infection					
	DNA^−^/AB^+^ (108)		DNA^+^/AB^+^ (27) *		DNA^−^/AB^−^ (256)	
	*n*	%	*n*	%	*n*	%
GENDER **						
Mare (380)	106	98.15	26	92.86	248	96.88
Stallion (10)	2	1.85			8	3.12
AGE						
3–6 years (78)	20	18.52	4	14.29	54	21.09
7–10 years (113)	30	27.78	5	17.86	78	30.47
11–15 years (129)	32	29.6	12	42.86	85	33.20
16–29 years (72)	26	24.07	7	25	39	15.23
**NUMBER OF BREEDING YEARS**						
Not covered (9)	2	1.85	2	7.14	5	1.95
1 breeding year (68)	17	15.74	2	7.14	49	19.14
2 breeding year (80)	17	15.74	5	17.86	58	22.66
3 breeding year (113)	29	26.85	10	35.71	74	28.91
4 breeding year (112)	41	37.96	9	32.14	62	24.22
**STOCK SIZE**						
1–9 horses (34)	4	3.70	4	14.29	26	10.16
10–39 horses (119)	35	32.41	9	32.14	75	29.30
>40 horses (239)	69	63.89	15	53.57	155	60.55
FOREIGN COUNTRY **						
Great Britain (27)	10	9.26	2	7.14	15	5.86
Ireland (33)	8	7.41	3	10.71	22	8.59
France (26)	8	7.41	3	10.71	15	5.856
Germany (227)	67	62.04	12	42.86	148	57.81
Other (4)	1	0.93	0	0	3	1.172
EqHV-COINFECTION **						
RNA^−^/AB^+^ (196)	60	55.57	12	44.45	124	48.44
RNA^+^/AB^+^ (41)	8	7.41	4	14.82	29	11.33
RNA^−^/AB^−^ (152)	40	37.04	10	37.04	102	39.84

* Data regarding potential risk factors could only be obtained for 27 of the 28 DNA^+^/AB^+^ horses. ** Data regarding gender, foreign country, and EqHV-coinfection could not be obtained for all the 392 horses.

**Table 2:** The newly identified specimens were submitted to the National Centre for Biotechnology Information (NCBI) and were assigned to the following accession numbers. The sample ID refers to Figure 2D–F.

Sample ID	EqPV Sequence Name	NCBI Accession Number
1	EqPV-H/VB1_518-1104nt	MN184860
3	EqPV-H/VB3_518-1104nt	MN184861
4	EqPV-H/VB4_518-1104nt	MN184862
10	EqPV-H/VB10_518-1104nt	MN184863
11	EqPV-H/VB11_518-1104nt	MN184864
17	EqPV-H/VB17_518-1104nt	MN184865
5	EqPV-H/VB5_518-1104nt	MN184866
19	EqPV-H/VB19_518-1104nt	MN184867
20	EqPV-H/VB20_518-1104nt	MN184868
23	EqPV-H/VB23_518-1104nt	MN184869
12	EqPV-H/VB12_518-1104nt	MN184870
14	EqPV-H/VB14_518-1104nt	MN184871
24	EqPV-H/VB24_518-1104nt	MN184872
25	EqPV-H/VB25_518-1104nt	MN184873
27	EqPV-H/VB27_518-1104nt	MN184874
15	EqPV-H/VB15_518-1104nt	MN184875

## Supplementary Materials

The following are available online at https://www.mdpi.com/1999-4915/11/10/965/s1, Figure S1: Correlation between EqPV-H and EqHV status of thoroughbreds. The EqPV-H status is displayed on the abscissa with the EqHV status shown in four shades of grey. Data for EqHV RNA and seroprevalence were obtained during a previous study [18]; Figure S2: Postnatal serum samples from a foal and an EqPV-H-positive mare were evaluated for the presence of EqPV-H VP1 antibodies and DNA at the indicated times using a luciferase immunoprecipitation (LIPS) assay and the relative increase of RLU compared to an EqPV-H-negative control sample was calculated.
