# Supplementary material for: Characterization of Equine Parvovirus in Thoroughbred Breeding Horses from Germany
Source: Viruses. 2019 Oct 18;11(10):965. doi: 10.3390/v11100965 (PMC6833105; doi:10.3390/v11100965)
Supplement: Supplementary file 1 [file viruses-11-00965-s001.pdf]

Supplement

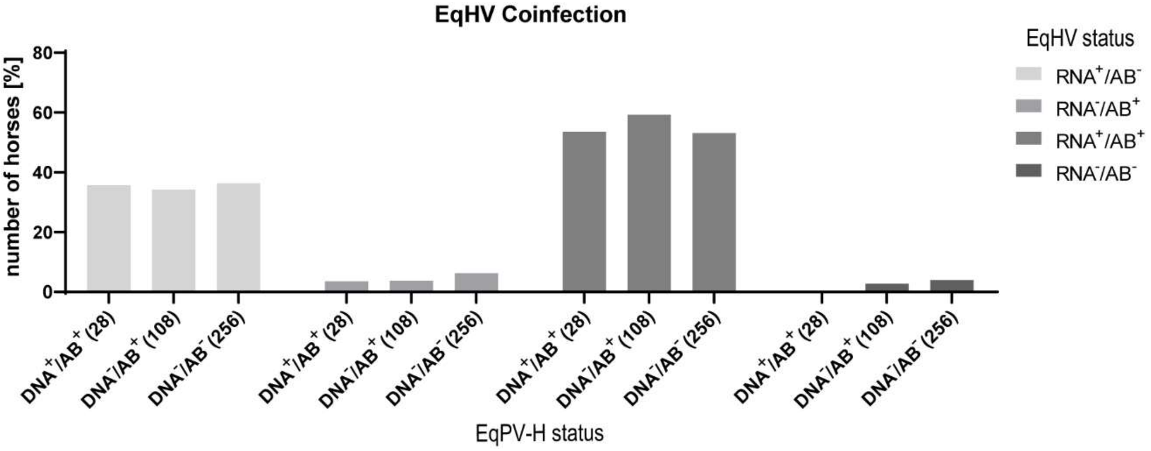

Supplementary Figure S1. Correlation between EqPV-H and EqHV status of thoroughbreds. The EqPV-H status is displayed on the abscissa with the EqHV status shown in four shades of grey. Data for EqHV RNA and seroprevalence were obtained during a previous study [18].

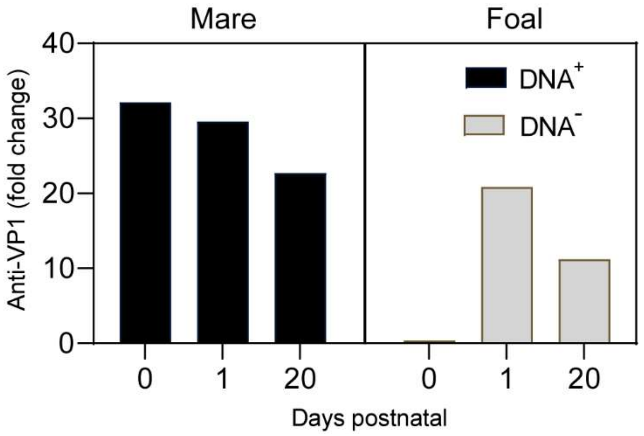

Supplementary Figure S2. Postnatal serum samples from a foal and an EqPV-H-positive mare were evaluated for the presence of EqPV-H VP1 antibodies and DNA at the indicated times using a luciferase immunoprecipitation (LIPS) assay and the relative increase of RLU compared to an EqPV-H-negative control sample was calculated.
